# Consequences of Team Job Demands: Role Ambiguity Climate, Affective Engagement, and Extra-Role Performance

**DOI:** 10.3389/fpsyg.2017.02292

**Published:** 2018-01-09

**Authors:** Miguel A. Mañas, Pedro Díaz-Fúnez, Vicente Pecino, Remedios López-Liria, David Padilla, José M. Aguilar-Parra

**Affiliations:** ^1^IPTORA Research Team, Department of Psychology, University of Almería, Almería, Spain; ^2^IPTORA Research Team, University of Almería, Almería, Spain; ^3^Hum-498 Research Team, Centre for Neuropsychological Evaluation and Rehabilitation, Department of Nursing Science, Physiotherapy and Medicine, University of Almería, Almería, Spain; ^4^Department of Psychology, University of Almería, Almería, Spain

**Keywords:** role ambiguity climate, affective engagement, extra-role performance, job demands-resources model, workplace well-being

## Abstract

In the absence of clearly established procedures in the workplace, employees will experience a negative affective state. This situation influences their well-being and their intention to behave in ways that benefit the organization beyond their job demands. This impact is more relevant on teamwork where members share the perception of ambiguity through emotional contagion (role ambiguity climate). In the framework of the job demands-resources model, the present study analyzes how high levels of role ambiguity climate can have such an effect to reduce employee affective engagement. Over time it has been associated with negative results for the organization due to a lack of extra-role performance. The sample included 706 employees from a multinational company, who were divided into 11 work teams. In line with the formulated hypotheses, the results confirm the negative influence of the role ambiguity climate on extra-role performance, and the mediated effect of affective engagement in the relationship between the role ambiguity climate and extra-role performance. These findings indicate that the role ambiguity climate is related to the adequate or inadequate functioning of employees within a work context.

## Introduction

When organizations establish procedures to increase the effectiveness and well-being of employees, the absence of ambiguity in the workplace is a key element in achieving success in such a venture. Among its main benefits is that employees have the capacity to plan, guide, and control the tasks they perform (Bieder and Bourrier, 2013). If in the context of work there are no clearly established procedures, employees must improvise their actions and their behavior will be based on their experience. This leads them to generate latent mistakes or produces direct negative consequences at the organizational level (Ramanujam and Goodman, 2003). If there is clarity in the procedures associated with a role, it increases the degree of accuracy with which the functions associated with that role are developed (Kahn et al., 1964).

Organizational climate *“emerges in organizations through a social information process that concerns the meaning employees attach to the policies, practices, and procedures they experience and the behaviors they observe being rewarded, supported, and expected”* (Schneider et al., 2013, p. 381). The clarification of climate as an attribute of the group or organization was an important step for climate research, although some researchers do continue to study climate at the individual level. Work team climate offers an approach to the tangibles on which managers can focus to generate the behaviors they require for effectiveness in the field of organizational studies and encourage a healthy organization (Salanova and Schaufeli, 2009; Schneider et al., 2013).

Role ambiguity is defined as the lack of clarity in understanding the actions to be taken to achieve proposed individual goals (Kahn et al., 1964). The existence of ambiguity with respect to objectives affects employees’ understanding of what they are expected to do, generates doubts about how to achieve their own performance objectives, and creates uncertainty as to how their performance will be assessed, and what the consequences will be for completing or failing to complete their objectives (Rogalsky et al., 2016).

The influence of role ambiguity on employees has been described as an affective state, which includes anxiety, depression, lack of self-confidence, or dysfunction in dealing with social situations (Lee and Ashforth, 1996). The consequences of role ambiguity, both at individual and team levels, have been analyzed in a multitude of studies, which underline the reduction of effort (Brown and Peterson, 1993; Tubre and Collins, 2000; Ortqvist and Wincent, 2006; Sakires et al., 2009; Doherty and Hoye, 2011) and decreased satisfaction (Jackson and Schuler, 1985; Thompson et al., 1997; Sakires et al., 2009).

When employees perceive ambiguity, the associated negative emotions will influence other partners due to emotional contagion (Hatfield et al., 1993). The context of the working group becomes the key to understanding how this perception is formed and will affect individuals (Haslam et al., 2003). Only a small number of investigations approach the research of stress from a work team level (Ehrhart, 2004; Peiró, 2008). Therefore, the study of organizational stress climate, including the climate of ambiguity, is currently receiving great interest to advance our understanding of the topic (Länsisalmi et al., 2000; Peiró and González-Romá, 2013).

Extra-role performance has been defined as actions not reflected in job descriptions that have an impact on increased well-being and organizational functioning (Bowling, 2010). In this way, we can differentiate in-role performance, which refers to actions that are “expected, evaluated and rewarded,” from extra-role performance, behavior that “arise spontaneously” (Leung, 2007, p. 45). Despite this differentiation, extra-role and in-role performance are strongly associated and extra-role behavior has been the focus of research in recent years (Hoffman et al., 2007; Caillier, 2016). Role ambiguity has shown to play an important role in extra-role performance. From the earliest research on this subject, the results have shown that employees will be more focused on their jobs after their role expectations have been clarified (Kahn et al., 1964). This suggests that when employees are unclear about their expected goals, they will put less effort into their jobs and will perform fewer behaviors that go beyond what is required by their contracts (Caillier, 2016). Chu et al. (2006) provide support for this assertion; they found that role ambiguity was negatively associated with employee willingness to display behavior that contributes to the social and psychological well-being of the organization.

The evaluation of work events as obstacles (i.e., role ambiguity) is related to the reduction of affective engagement, which results in decreased employee well-being (Cavanaugh et al., 2000; Crawford et al., 2010; Schaufeli and Taris, 2014). Kahn (1990, p. 694) introduced the concept of engagement, conceptualizing it as the *“harnessing of organization members’ selves to their work roles; in engagement, people employ and express themselves physically, cognitively, and emotionally during role performances.”* Affective engagement, as an indicator of well-being, is defined as the experimentation of a state of positive affect related to the work role itself. This process is characterized by an increase in an individual’s physical, cognitive, and emotional effort in developing their work (Soane et al., 2012), and has a beneficial effect on thought processes (Fredrickson, 1998) and individual activation (May et al., 2004; Macey and Schneider, 2008).

Several studies have found that the affective bond between the worker and the organization is very sensitive to job demands such as role ambiguity. Jackson and Schuler’s (1985) meta-analysis found a significant correlation between role ambiguity and negative affective responses in employees. Recently, research in role ambiguity such as O’driscoll and Beehr (2000), Adae et al. (2008), Brunetto et al. (2011), Caillier (2012), and Chenevert et al. (2013) has determined its significance and negative influence on the affective status of several groups. Recent studies have found that affective engagement also shows a significant mediating effect on organizational outcomes and working environments in their different conceptualizations (Yalabik et al., 2013; Schmitt et al., 2016).

Previously, other authors have sought to understand the behavior of variables in the organizational context by explaining that it is necessary to study complex explanatory models to describe the relationships between these variables (Ehrhart, 2004; Ferris et al., 2009). The job demands-resources (JD-R) model provides a suitable frame of reference for researching the mediating effects in several levels of analysis (Bakker and Demerouti, 2014; Bakker et al., 2014). This assumes that any job has associated factors that influence employee well-being through two processes: a health impairment process and a motivational process. In health impairment process, job demands are an important predictor that reduces employee engagement, and over time is associated with negative results for the organization (Bakker et al., 2014). The importance of the JD-R model in the context of employee stress or performance has been supported by studies in different countries and occupational groups (Demerouti et al., 2001; Bakker et al., 2003; Salanova et al., 2005; Ceschi et al., 2017b), and the model has been successfully adapted to multilevel analysis studies (Bakker et al., 2003; Llorens et al., 2006). Recently, authors as Ceschi et al. (2016) have examined the role of two personality traits (grit and honesty–humility) in the health impairment process and counterproductive work behavior.

In the framework of the JD-R model, the present study analyzes how the role ambiguity climate is an important predictor for reducing employee affective engagement in terms of job demands, and over time is associated with negative results for the organization due to a lack of extra-role performance.

Thus, we hypothesize that the role ambiguity climate has a negative effect on employee affective engagement and extra-role performance. In addition, affective engagement mediates the relationship between the role ambiguity climate and extra-role performance.

## Materials and Methods

### Participants

The final sample of this study comprised 706 employees from a multinational private service sector company based in Spain. The participants’ ages were distributed in five intervals (up to 25 years = 22.6%, 26–35 years = 31.2%, 36–45 years = 29.8%, 46–55 years = 15%, and 56 or more years = 1.4%). The sample included 91.2% males and 8.8% females. Regarding the contractual relationship, 4.4% had a temporary part-time contract, 28% had a full-time temporary contract, 3% had an indefinite part-time contract, 62.6% were hired in a full-time indefinite capacity, and 2% had another type of contract. Most of the sample had higher general secondary education or vocational training (59.4%), 22.7% had a bachelor degree, 11% had a university degree, and others made up the remaining 6.8%. These employees were grouped into 11 work teams (between the administrative office sector, general service assistance, and company support services), with an average team size of 49.2 workers (*SD* = 21.2).

### Procedure

This is a descriptive, cross-sectional study in which data were collected through questionnaires. The Ethical Review Committee at the University of Almería approved the study. The procedure for collecting information began with holding several meetings with those responsible for the company. The questionnaire application was an anonymous form that was completed electronically in the work center, and all workers previously signed an informed consent form. During the application, a member of the research group remained in the room to address any doubts that participants might have about filling the questionnaires. Thus, 100% of the questionnaires were completed correctly and could be used for further analysis.

### Instruments

**FIGURE 1:**
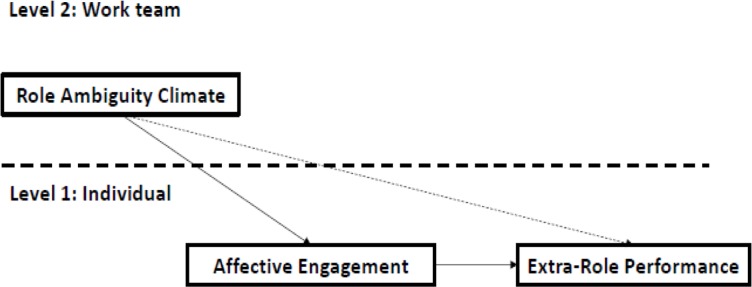
The job demands-resources model applied to the hypothesis model.

This paper adapts the JD-R model to the study of the perception of role ambiguity (as a job demand) analyzed at the level of teamwork, and the mediated effect of the affective engagement between extra-role performance and role ambiguity (**Figure 1**).

#### Role Ambiguity

It has been measured through the questionnaire of Rizzo et al. (1970) adapted by Peiró et al. (1986). The response format is a Likert type of five response alternatives ranging from 1 (“strongly disagree”) to 5 (“strongly agree”). It is composed of six items (i.e., “I know clearly what my responsibilities are”) and has a Cronbach’s alpha coefficient of 0.89. The corrected item-total correlation ranged from 0.55 to 0.77. When exceeding the value of 0.40, it can be concluded that these items are reliable and show their validity (Stewart and Ware, 1992).

#### Affective Engagement

It was measured with the Spanish version of the Intellectual, Social, Affective Engagement Scale of Soane et al. (2012) adapted by Mañas et al. (2016). Affective engagement is measured by three items (i.e., “I feel full of energy and strength with my work”). Cronbach’s alpha for this scale was 0.94. A Likert scale with seven categories ranging from 1 (“strongly disagree”) to 7 (“strongly agree”) was used for their response. The corrected item-total correlation ranged from 0.58 to 0.77.

#### Extra-Role Performance

It was measured using the dimension contextual performance of Job Performance Scale of Goodman and Svyantek (1999). The scale consists of seven items (i.e., “Helps other employees with their work when they have been absent”). The internal consistency of the scale was 0.93. Participants had to answer a 7-point scale ranging from 1 (“strongly disagree”) to 7 (“strongly agree”). The corrected item-total correlation ranged from 0.65 to 0.71.

### Statistical Analysis

#### Aggregation Index

Role ambiguity has been analyzed as a predictor at the level of the work teams that make up the company. For this, it is necessary to evaluate the degree of agreement in the perceptions of the different members that compose these teams. The ICC1 and ICC2 indices were calculated for this purpose (Bartko, 1976). Although there is no preset fixed cutoff point, for the ICC1 index a value of 0.01 could be considered as a small effect, a value of approximately 0.10 could be considered as an average effect, and values greater than 0.25 could be considered as a large effect (Lebreton and Senter, 2008). For ICC2, values above 0.60 would show support for aggregation. From a consensus-based approach, the Average Deviation Index [ADM(J)] (Burke et al., 1999) and Rwg(J) (James et al., 1993) have been used, and it was concluded that there is a consensus in the unit when the ADM(J) was equal to or less than 1 (Burke et al., 1999). The Rwg(J) indicates elevated levels of aggregation in the work teams when it shows values greater than 0.70 (Lance et al., 2006). Analysis of variance (ANOVA) has been used in order to determine if there was significant discrimination between the scores displayed by the different groups.

The ICC1 and ICC2 indices obtained for the role ambiguity variable were 0.56 and 0.82, respectively. The average value of ADM(J) was 0.89, while the value of Rwg(J) was 0.69. The results of the ANOVA analysis have shown statistically significant differences that support the discrimination between the teams that compose the sample for the analysis of role ambiguity, *F*(26,411) = 2.60, *p* < 0.001. These aggregation results indicate the adequacy of the aggregation of the values of the role ambiguity variable between the units that compose the studied sample.

#### Linear Hierarchical Multilevel Model

To test the hypotheses, a hierarchical linear model was used (Hofmann et al., 2000), including a mediator variable at the individual level (affective engagement, model 2-1-1). Zhang et al. (2009) argue that, since members of the organization are grouped into work teams, testing the proposed mediation effects by using traditional procedures will result in biased coefficients. Therefore, we followed the procedure recommended by these authors to test the effects of mediation in multilevel contexts.

The effects of mediation can be estimated erroneously when different values are obtained inside and outside the group with respect to magnitude. Therefore, although hierarchical models of mediation can be tested using the traditional Sobel (1982) procedure, more caution is needed. In the present study, we used the analysis of hierarchical models as described in the paper by Zhang et al. (2009). Therefore, the predictor scores have been centered on the mean of the group, and the mean has been included in the level-two interception equation [called CWC (M) or centered within context with the reintroduction of the subtracted means]. This procedure also allows a more accurate test of cross-effects and reduces the problems of estimation at the aggregate level of analysis (Hofmann and Gavin, 1998; Raudenbush and Bryk, 2002). Once we obtained each coefficient of the corrected models, mediation was tested using the Sobel test’s step-by-step approach (Baron and Kenny, 1986).

## Results

Mean, standard deviation, internal consistency, and correlations between the variables are shown in **Table 1**. All correlations were significant and showed the expected pattern of interrelations between the study variables. The role ambiguity climate correlated negatively and significantly with affective engagement (*r* = -0.34, *p* < 0.001) and with extra-role performance (*r* = -0.20, *p* < 0.001). On the other side, there was a significant and positive correlation between extra-role performance and affective engagement (*r* = 0.34, *p* < 0.001).

**Table 1:** Means, standard deviations, internal consistencies, and correlations.

Variables	*M*	*SD*	1	2	3	4
(1) Role ambiguity climate	1.79	0.81	(0.89)			
(2) Affective engagement	5.95	1.10	-0.39^∗∗∗^	(0.94)		
(3) Extra-role performance	6.03	0.92	-0.24^∗∗∗^	0.34^∗∗∗^	(0.93)	
(4) Age	2.42	1.04	–	–	–	(-)

*Internal consistencies over the main diagonal. ^∗^*p* < 0.05; ^∗∗^*p* < 0.01; ^∗∗∗^*p* < 0.001*.

The hierarchical regression model (**Table 2**) shows the mediating effect of affective engagement on the relationship between the role ambiguity climate and extra-role performance. First, the results in **Table 2** show the negative and significant influence of the role ambiguity climate and extra-role performance (β = -0.20, *p* < 0.001), and a negative and significant influence of the role ambiguity climate and affective engagement (β = -0.35, *p* < 0.001). None of the two outcome variables influence the age variable which was used as a control variable (affective engagement: β = 0.04, *p*, ns; extra-role performance: β = -0.03, *p*, ns). The second step of the regression model includes the mediating effect of affective engagement in the regression equation. The results show that role ambiguity climate and extra-role performance are significantly reduced (β = -0.9, *p* < 0.05) in step 1. The non-significant effects of age are maintained. These results confirm the partial mediation of affective engagement between role ambiguity on teamwork and extra-role performance.

**Table 2:** Results for the hierarchical regression models.

	Mediator		Extra-role performance	
Step and variable	β	*SE*	β	*SE*
(1) Age	0.04	0.03	-0.03	0.03
Role ambiguity climate	-0.34^∗∗∗^	0.06	-0.20***	0.05
(2) Age			-0.04	0.03
Role ambiguity climate			-0.09*	0.06
Affective engagement (*M*)			0.31***	0.03

*M, mediator. ^∗^*p* < 0.05; ^∗∗^*p* < 0.01; ^∗∗∗^*p* < 0.001*.

## Discussion

The objective of this study has been to analyze the effects of role ambiguity on work teams, affective engagement, and the extra-role performance of employees, studying if affective engagement mediates the relationship between them. For this purpose, the JD-R model (Bakker and Demerouti, 2014) has been used as a basis for the validation of these hypotheses.

Our first hypotheses argued that role ambiguity would have a significant and negative effect on affective engagement and extra-role performance. The results have confirmed these associations, and suggest that the existence of elevated levels of ambiguity in work teams will reduce affective engagement among employees and extra-role performance behaviors that are carried out in the workplace. Other authors also have found that employees who perceive ambiguity in the definition or execution of their functions experience lower levels of effort (Adae et al., 2008; Moliner et al., 2008; Brunetto et al., 2011; Caillier, 2012). Therefore, the purpose of the clarification of employee roles is the facilitation of the fulfillment of objectives associated with each job, which can have positive effects on the health and well-being of employees (Martínez-Córcoles et al., 2014).

The other hypotheses raised the effect of mediation of affective engagement on the relationship between role ambiguity climate and extra-role performance. Thus, when affective engagement is included in the regression equation, the influence of role ambiguity climate in extra-role performance reduces its influence considerably (β = -0.9, *p* < 0.05). This suggests that the existence of a role ambiguity climate in the work teams influences extra-role performance, but mainly through its impact on affective engagement. Previous literature has described role ambiguity’s influence on effectiveness, satisfaction with reward for self-effort, and engagement (Ortqvist and Wincent, 2006).

Therefore, extra-role performance is positively influenced by the combination of clear roles in work teams and affective engagement. This is because such employees are able to better manage their existing work resources. In order to achieve their goals, organizations need their employees to personally commit to the achievement of collective goals (Rodríguez-Montalbán et al., 2014). The behavior associated with these collective goals have been denominated in several ways, for example, by extra-role performance (Walumbwa et al., 2010).

This research has considered role ambiguity from a multilevel perspective in line with previous work on stress climates (Ehrhart, 2004; Peiró, 2008; Peiró and González-Romá, 2013; Kozusznik et al., 2015). It proposes the JD-R model as a starting point for the study of the influence of role ambiguity on work teams. This would assist in understanding the variation in worker perceptions and affective engagement perspectives, which would influence effort levels in developing behavior oriented toward extra-role performance. Previous research (Chu et al., 2006; Caillier, 2016) showed, without introducing the affective engagement variable, how role ambiguity affected extra-role performance by analyzing the former from an individual level. In current psychosocial research literature, work team climate is an essential element in the field of organizational studies (Schneider et al., 2013, 2017).

### Limitations and Future Research Directions

The results obtained in this study should be considered under the following limitations. First, the results were obtained from self-reports and could be affected by the variance of the common method; however, the use of intersubjective responses at the team level (aggregate role ambiguity responses) could mitigate this effect. Second, the sample is very specific, and is limited to the collective personnel of a multinational organization located in Spain. Therefore, the generalizability of the results of this study is limited, and they should be considered with this caveat in mind. Despite these limitations, the results are very relevant to further our knowledge of the variables that can help to improve the psychological well-being of employees (in order to obtain inputs for interventions) and to develop healthier organizations. The last limitation is with regard to the cross-sectional study, which does not allow for the observation of causality in the relationships between predictors and outcomes by controlling for stabilities.

Future research should examine the use of other forms of data collection, in addition to self-reported tools (as direct observation or critical incident evaluation interviews). This would provide complementary measures that would corroborate the validity of the data. Second, it is convenient to increase the study sample spectrum in other workplaces, which will allow for a comparison of the results in other work contexts. Of relevance in future research will be the comparison of samples of private and public administrations (Costantini et al., 2017). Third, it is necessary to carry out longitudinal studies that will allow us to analyze the evolution and causal influences, for example, in improving the perception of justice about well-being, group performance, and work–family balance. In addition to the proposed changes based on the limitations of this work, it would also be advisable to deepen the study of the antecedents of the perception of affective commitment in employees. Leadership styles, diverse types of work climates, and other variables can act as filters in obtaining different perceptions of affective commitment in a certain job.

### Practical Implications

The results of the study have important implications for staff management in organizations with shared perceptions of role ambiguity in work teams (as a stress climate). The way in which the perception of role ambiguity is managed is the determinant for achieving employee well-being and engaging all employees, not only those with affective engagement (Soane et al., 2012). The effectiveness of the organization will improve by having employees who are more willing to perform functions beyond those who are described in their job descriptions (extra-role performance). At this point, it is convenient to remember that in situations of high labor demands, it is necessary for employees to commit themselves personally and affectively to the achievement of the proposed collective objectives (Rodríguez-Montalbán et al., 2014). The effectivity of the occurrence of this type of behavior has been observed in many studies (MacKenzie et al., 1991; Van Dyne et al., 1995; Vandaele and Gemmel, 2006). These measures will, in turn, promote psychosocial health within organizations and increase employee resilience levels (Meneghel et al., 2016), making public administration a healthy organization (Costantini et al., 2017; Mañas and Alcaraz-Pardo, 2017). In this approach, researchers try to discuss the role of different organizational strategies to preserve an organization’s health (Ceschi et al., 2017a).

Finally, the organizations interested in enhancing employee extra-role performance and affective engagement must define their employees’ functions or tasks with more comprehensive performance information. This means that employees can proactively use and seek information, initiate a manual structure, and ultimately, when the organization facilitates actions by clarifying, planning operations, communicating changes, and monitoring activities through effective leadership, these activities can help to improve role clarity (Kohli, 1989; Brown et al., 2001; Hall, 2008; Yukl et al., 2009).

## Author Contributions

MM, PD-F, and VP: contribution to the conception and design of the work; the acquisition and interpretation of data for the work, revising it critically for important intellectual content and final approval of the version to be published. RL-L, DP, and JA-P: contribution to the conception and design of the work; the interpretation of data for the work, revising it critically for important intellectual content and final approval of the version to be published. All authors are accepting and agreeing that the work is original; any methods and data presented are described accurately and honestly; any relevant interests have been disclosed.

## Conflict of Interest Statement

The authors declare that the research was conducted in the absence of any commercial or financial relationships that could be construed as a potential conflict of interest. The reviewer AC and handling editor declared their shared affiliation.
